# Combination of a bronchogenic cyst in the thoracic spinal canal with chronic myelocytic leukemia

**DOI:** 10.1515/biol-2022-0736

**Published:** 2023-09-30

**Authors:** Hao Zhang, Hai-Feng Li, Hai-Feng Duan, Ke-Feng Huang, Zhi-Hua Tian

**Affiliations:** Department of Neurosurgery, Jin Cheng People’s Hospital, No. 1666 Baishui East Street, Jincheng, Shanxi 048026, China

**Keywords:** bronchogenic cyst, case report, intramedullary, chronic myelocytic leukemia, surgical resection

## Abstract

The presented case report describes an incredibly rare instance of an intramedullary bronchial cyst located in the thoracic spinal canal on the dorsal side of the spinal cord, which was observed in a patient with chronic myelogenous leukemia. A 29-year-old man presented with back pain for half a month, along with numbness and pain below the chest and ribs for 1 week. Hypersensitivity was present in the inferior plane of the long xiphoid process in the nervous system. Magnetic resonance imaging (MRI) showed intramedullary cystic lesions in the vertebral body plane of the third to the fourth thoracic vertebra. There was no recurrence during the 6-month postoperative follow-up period. The histopathological findings were consistent with bronchogenic cysts. Cystic lesions were eliminated through the posterior median approach. After the cyst ruptured during surgery, gel liquid was seen, and the majority of the cyst walls were removed. One week after the surgery, the hypersensitivity fully subsided. Six months following surgery, an updated MRI revealed no recurrence. Intramedullary bronchogenic cysts on the dorsal side of the thoracic spine are extremely uncommon. Diagnosis requires histopathological evidence, and it is challenging to diagnose before surgery. Prompt surgical resection is recommended in case of positive diagnosis.

## 1 Introduction

Bronchogenic cysts are cystic lesions that develop due to abnormal bronchial system development during the embryonic stage. It is an endodermal translocation tumor that frequently develops in the external spinal membrane of the cervical and thoracic spinal cords. Most of the sites are located in the ventral spinal cord. It rarely occurs in the spinal canal, and accounts for 10–15% of mediastinal masses. The first instance of an intraspinal bronchogenic cyst was reported by Yamashita in 1973 [1]. It is incredibly uncommon for cysts to occur anywhere else besides the cervical and thoracic spine. A few cases of intraspinal bronchogenic cysts have been recorded in China over the past several years [2]. However, it should be noted that locating such a cyst in the thoracic spinal cord, as seen in the current case, is extremely rare.

## 2 Medical record reporting

### 2.1 History and physical examination

A 29-year-old male patient was admitted to the Jincheng People’s Hospital, Shanxi Province, with back pain for half a month and numbness and pain below the chests and ribs for 1 week. A thorough physical examination was conducted upon admission. The patient was able to move his limbs based on instructions and could maintain autonomous postures. He had no swelling of superficial lymph nodes in the whole body, showed hypersensitivity below the xiphoid plane, and there was a symmetrical degree of hypersensitivity on both sides. The muscle strength of both lower limbs was grade V on the Lovett scale, the muscle tension was normal, the muscles of both lower limbs were not atrophied, and the pathological symptoms of both lower limbs were not induced. Past medical history: the patient was diagnosed with chronic myelocytic leukemia in the chronic stage in the Department of Hematology, Jincheng People’s Hospital in July 2016. The patient was orally administered imatinib mesylate tablets 400 mg/per time per day. Regular blood tests detected no abnormalities in the white blood cells or platelets. There was an intraspinal cystic lesion with hemivertebra deformity in the region of the third to the fourth thoracic vertebra on plain and enhanced magnetic resonance imaging (MRI) scans of the thoracic vertebra.

**Figure 1:**
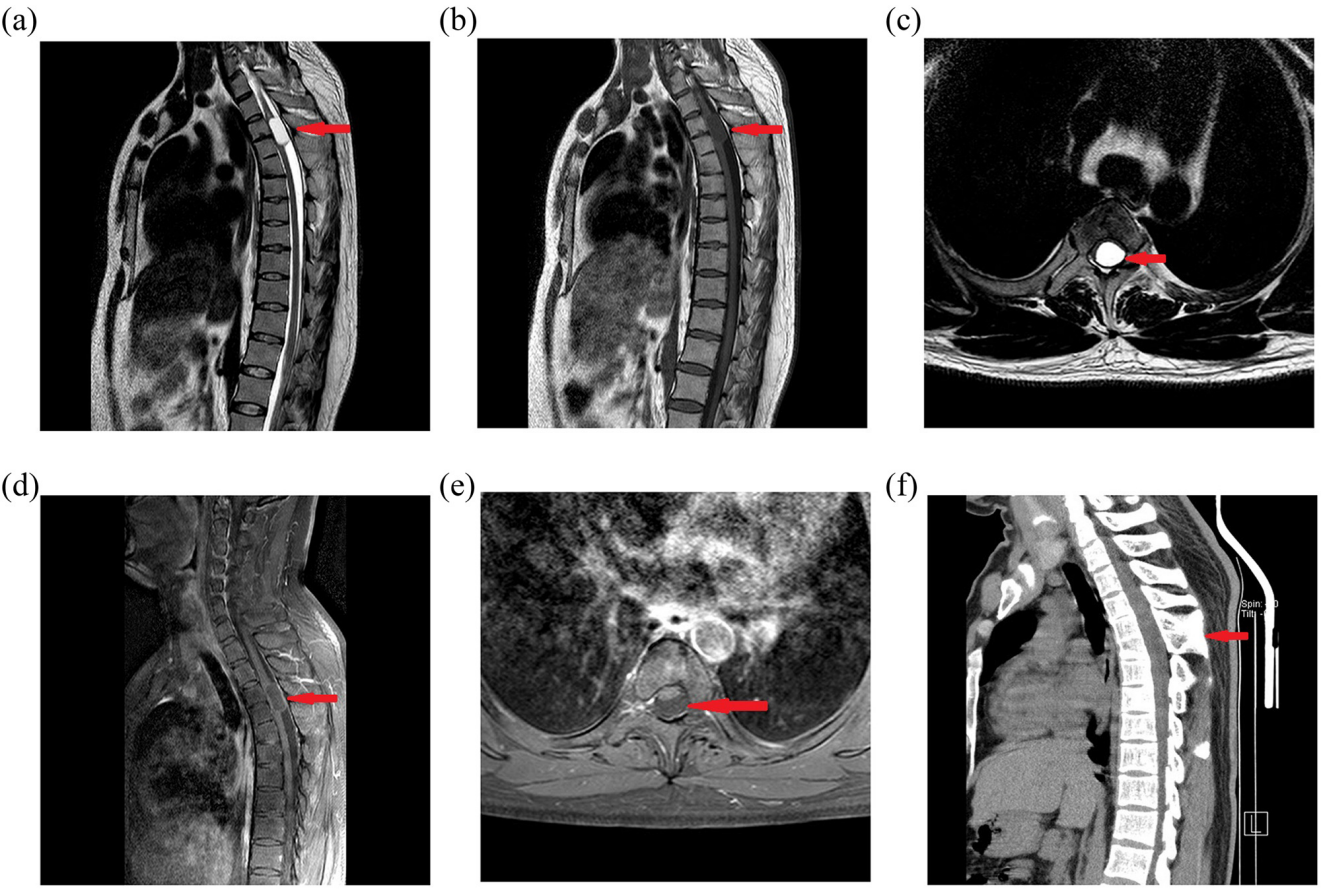
(a) T2 image of a sagittal plain MRI scan. On the third to the fourth thoracic vertebral plane, a cystic mass with significant signal intensity could be identified. It is evident that the spinal cord is compressed. (b) T1 image of a sagittal plain MRI scan. On the third to the fourth thoracic vertebral plane, a cystic mass that is T1 isointense or slightly hypointense is visible. (c) A horizontal T2 image from a plain MRI scan. The left dorsal side of the spinal cord has a cystic mass, and the spinal cord is compressed. (d) Enhanced sagittal plain MRI image. An enhanced cystic mass can be seen. (e) Enhanced sagittal plain MRI image. There is no visible cystic space occupying the enhancement. (f) Sagittal plain CT image showing the third to the fourth thoracic vertebrae and spinous process fusion.

**Figure 2:**
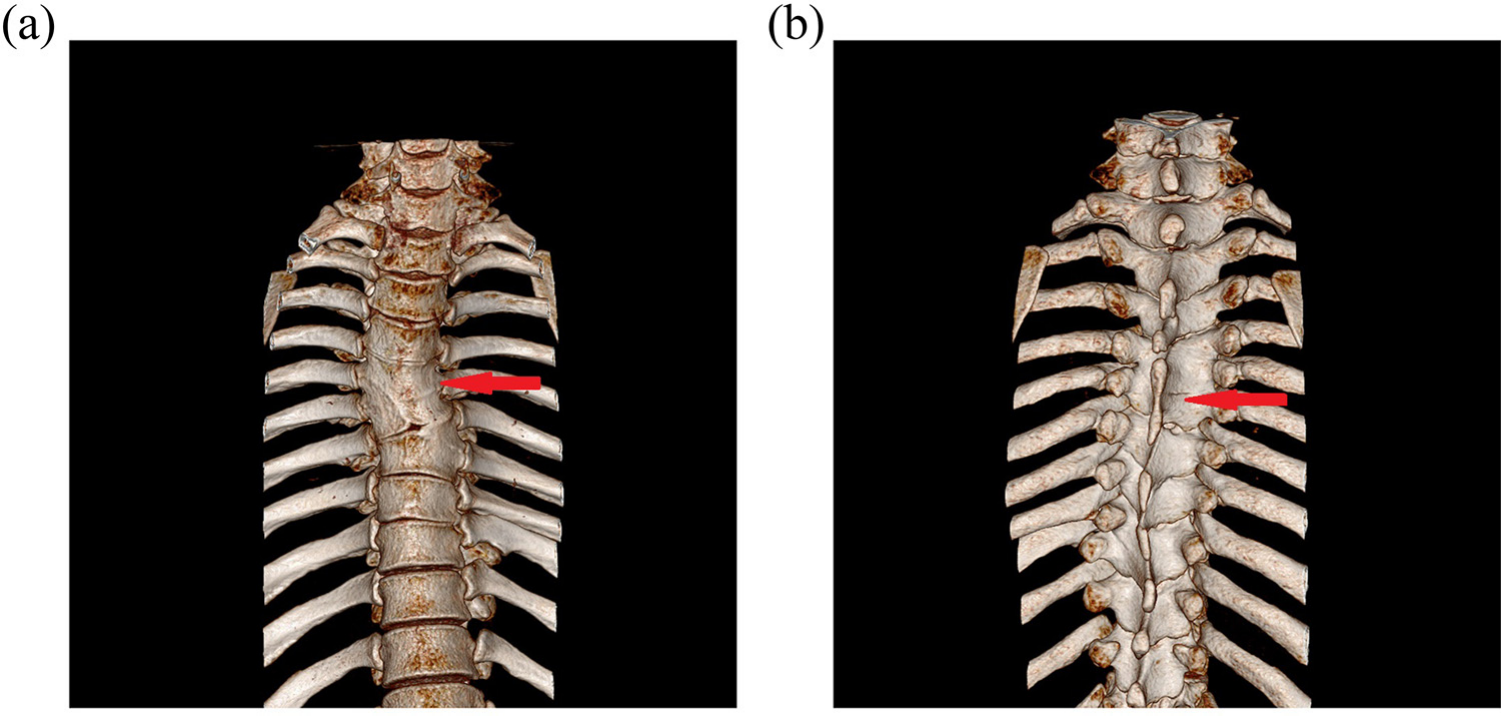
(a) and (b) CT three-dimensional reconstruction of the third to the fourth thoracic vertebrae with spinous process fusion.

The plain thoracic vertebra MRI scan is displayed in Figure 1a–c. The result showed that at the thoracic 3–4 vertebral body plane, T1 signal and T2 high signal can be seen in the cystic space, the spinal cord compression was severe, and the cystic fluid signal was homogeneous. The cyst wall was smooth. The enhancement of cyst wall was not obvious in enhanced scan. The enhanced thoracic vertebra MRI scan is displayed in Figure 1d and e. The plain thoracic vertebra computed tomography (CT) scan is displayed in Figure 1f; and the three-dimensional reconstruction is displayed in Figure 2a and b. CT showed malformations of fusion of spinous processes of thoracic 4 and thoracic 5.

The initial diagnosis was a space-occupying lesion in the spinal canal of the third to the fourth thoracic vertebra.


**Informed consent:** Informed consent has been obtained from all individuals included in this study.
**Ethical approval:** The research related to human use has been complied with all the relevant national regulations, institutional policies and in accordance with the tenets of the Helsinki Declaration, and has been approved by the Ethics Committee of Jin Cheng People’s Hospital (JCPH No. 20230629001).

### 2.2 Surgery and pathology

**Figure 3:**
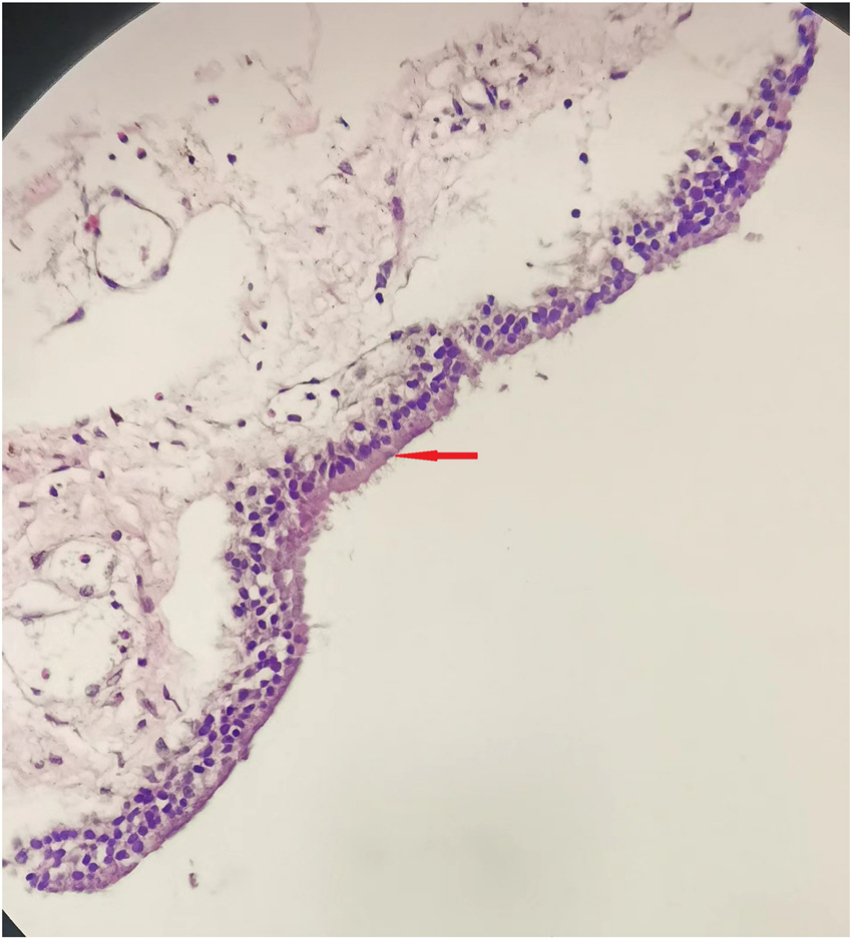
HE staining of a pathological section at 40×. The cyst wall is made of columnar epithelial cells.

Cyst lesions were surgically resected using a posterior midline approach. During the surgery, a grinding drill is used to remove the entire vertebral lamina and spinous process, and the dura mater is incised. The partial vertebral plate and spinous process were also resected during the procedure, which also revealed a cystic tumor in the dorsal side of the spinal cord of the third to the fourth thoracic vertebra. There was no spinal cord pulse after the dura mater spinalis was incised. A fine needle was inserted into the cyst, which produced a gel-like fluid. The tumor was entirely resected under a microscope and the cyst wall was thin, grayish white, and translucent (Figure 3). On the second day after surgery, the symptoms of chest and back pain significantly disappeared. The patient did not experience any sensory or motor impairment in both legs, nor did he have any bowel or bowel movements. There were no complications. The examination images indicate that the tumor capsule conforms to the ciliated columnar epithelial structure of the bronchial wall. Postoperative examination showed an intraspinal bronchogenic cyst.

### 2.3 Postoperative follow-up

**Figure 4:**
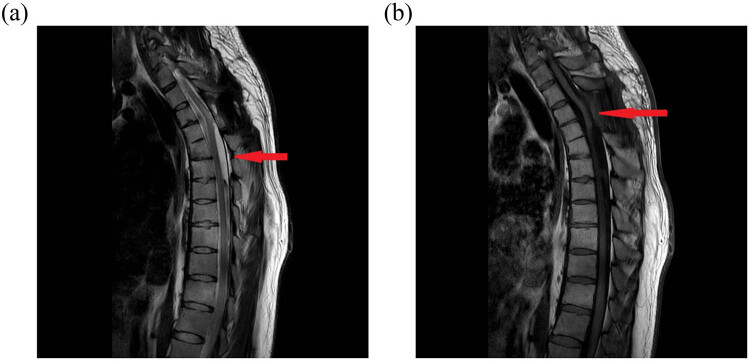
(a) T2 images from a sagittal plain MRI scan re-examined 6 months following surgery. The cystic space that was occupied vanished without a trace. When compared to before surgery, the spinal cord morphology is dramatically improved. (b) T1 images of sagittal plain MRI scan re-examined 6 months following surgery. Cystic space that was occupied vanished without recurrence. When compared to before surgery, the spinal cord morphology is dramatically improved.

The patient’s hypersensitivity was eliminated 1 week after the operation, and there was no sensation or movement disorder in his limbs. Following a complete MRI, no evident recurrence was discovered 6 months after the operation (Figure 4).

## 3 Discussion

Intraspinal bronchial cysts are a rare congenital developmental anomaly of the respiratory system that arise from the pre-embryonic intestine. They account for approximately 0.7–1.3% of all intramedullary spinal cord tumors. The surface of the cyst is covered by respiratory epithelium [3]. There are currently three theories put forth in literature that attempt to explain the exact pathogenesis of bronchial cysts. (1) Isolation insufficiency hypothesis: insufficient isolation occurs when the endoderm and ectoderm are being differentiated into separate cell masses [4,5]. (2) Potential differentiation hypothesis: due to the capacity of the ectoderm to differentiate into endoderm and paraxial mesoderm, it is believed that cysts originate from the ectoderm [6]. (3) Split notochord syndrome: this condition is thought to be caused by incomplete replication or separation of the notochord, which could explain the local defect. This hypothesis can explain the phenomenon that bronchogenic cysts in the spinal canal often combined with other malformations [7,8]. Another abnormal differentiation hypothesis has been added in Chinese literature – bronchogenic cysts originate from abnormal differentiation of the tracheobronchial tree [9]. The notochord fissure syndrome is the currently preferred hypothesis. However, the specific pathogenesis of this disease still needs to be further clarified by accumulating a large amount of case data. In this case, the condition of the patient with the combined spinal malformation was in concordance with the hypothesis of the split notochord syndrome.

Clinical manifestations and symptoms of intraspinal bronchial cysts are generally site-related and non-specific. Neck and back pain, as well as sensory and motor abnormalities in the four limbs are the most typical symptoms. In this case, back and chest pain were the main symptoms of the patient.

On MRI, the majority of intraspinal bronchogenic cysts show hypointense T1 and hyperintense T2 signal, and gadoteric acid meglumine does not clearly augment these signals. As it can be challenging to discern one condition from another using an MRI alone, a CT scan is required. Spina bifida and other congenital spinal abnormalities can be seen on a CT scan [10].

Pathologically, intraspinal bronchogenic cysts are mostly characterized by the single-layer or pseudostratified columnar epithelium of the cyst wall, and glands, cartilage, smooth muscles, and nerve fibers may be present on the cyst wall. The fluid within the cyst is composed of various substances, with most exhibiting a gel-like or yellowish appearance. However, some portions of the fluid may be clear and colorless.

The primary form of treatment for an intraspinal bronchogenic cyst is surgery. During the procedure, the spinal cord and nerve root should be safeguarded, and the total cyst wall resection should not be forced. It is reported that the cyst wall may be washed with diluted iodophor or ignited by local bipolar electrocoagulation micro-current after the cyst wall has been incised to liberate the cyst fluid [11]. Additionally, subtotal resection is also acceptable. According to published data, 84.6% of patients with intraspinal bronchogenic cysts who underwent subtotal resection experienced no recurrence at the end of the follow-up [12]. However, this study also recommended total resection, the recurrence rate after subtotal resection was 15.4%, but there was no recurrence after total resection. In addition, incomplete excision, cystic fenestration, and biopsy associated with recurrence should be avoided [12]. In this case, the cyst wall was completely resected during the operation, and there was no postoperative recurrence. This result was consistent with the conclusions of the above studies.

The patient in our study had a bronchogenic cyst of the spinal canal complicated with chronic myelogenous leukemia. The preoperative surgical plan fully estimated the possibility of intraoperative bleeding, the number of white blood cells, red blood cells, platelets, and the coagulation function of the patient should be paid attention to in preoperative detection. As we all know, patients with leukemia often have coagulation dysfunction, resulting in excessive intraoperative field bleeding, and continuous postoperative bleeding leads to epidural or subdural hematoma, resulting in lower limb dysfunction [13]. Therefore, it is necessary to prepare enough blood before operation, hemostasis during operation should be thorough, and blood routine and coagulation function should be reviewed after operation too. In addition, the patient’s systemic condition, including whether there is ecchymosis and bleeding, as well as lower limb function, should be carefully observed. Once lower limb dysfunction occurs timely surgery is required to remove hematoma.

## 4 Conclusion

Intraspinal bronchogenic cysts, especially those affecting the dorsal thoracic region, are extremely rare, and the case described in this report falls within this uncommon category. To confirm the diagnosis of this condition, imaging and clinical pathology examinations are essential. Surgery is generally considered the preferred method of treatment.
